# Salvage multivisceral abdominal surgery after caustic ingestion: Case report

**DOI:** 10.1016/j.ijscr.2020.05.007

**Published:** 2020-05-19

**Authors:** Cristina Maggioni, Luca Voltolini, Stefano Bongiolatti, Fabio Cianchi, Francesco Coratti

**Affiliations:** Center for Oncological Minimally Invasive Surgery (COMIS), Department of Experimental and Clinical Medicine, University of Florence, Largo Brambilla 3, 50134, Florence, Italy

**Keywords:** Case report, Caustic ingestion, Caustic lesions, Suicide attempted, Emergency surgery, Multi-visceral surgery

## Abstract

**Introduction:**

Ingestion of caustic substances is a life-threatening medical emergency with high morbidity and mortality rate.

**Case report:**

We present a case report of young patient who presents to our department secondary to ingestion of caustic substances was submitted to multi-visceral resection surgery having saved his life after a long and complex multidisciplinary management.

**Conclusion:**

An aggressive surgical approach allows successful initial treatment of extended caustic injuries. Early surgical treatment is essential to improve the prognosis in these patients and refer the patient to large and experienced hospital represents the main goal to guarantee a surviving chance.

## Highlights

• Caustic ingestion represents a drama for patients and doctors.
• Emergency treatment is hard and needs experienced team.
• Despite is mainly related to suicide attempt our goal is to guarantee the patient’s survival.
• This report represents a prompt management with II step reconstruction.
• Multi-disciplinary approach with high competency represents the only possibility to manage a so complex situation.

## 1 Introduction

Ingestion of caustic substances is a life-threatening medical emergency with high morbidity and mortality rate. The real incidence is currently unknown and largely underreported. The most commonly ingested caustic are acidic or alkaline substances [1]. The poison control center of the United States reported more than 200,000 annual exposures by ingestion of toxic substances [2]. These injuries may be encountered both in adult and pediatric patients, with children representing 80% of ingestion injuries; however, the pattern of injury differs significantly. In children younger than 5 years, 80% of caustic ingestion occurs accidentally while the adults usually ingest caustic substances as an attempted suicide. Approximately 1–2% of case in all age groups results in stricture formation and 10% of cases in adult result in death [3]. Identification of the nature, the physical form, and the quantity of the ingested agent as well as the accidental voluntary ingestion pattern are the cornerstones for emergency management of corrosive injuries [[4], [5], [6]]. We present a case report of young patient who presented to our academic department secondary to ingestion of caustic substances treated with multi-visceral resection surgery. The work has been reported in line with the SCARE criteria [7].

## 2 Case report

**Fig. 1:**
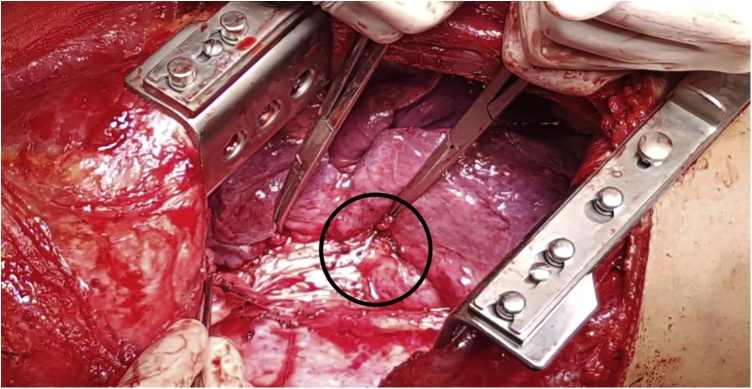
Esophagobronchial fistula (in black circle).

**Fig. 2:**
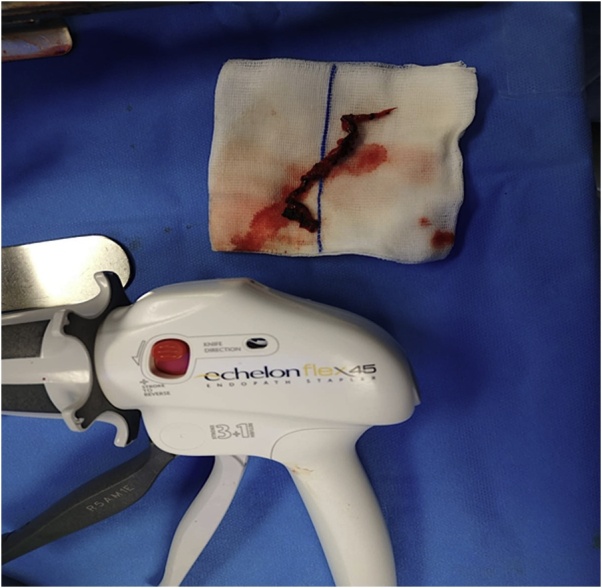
Resected esophagus (note the size and the length).

**Fig. 3:**
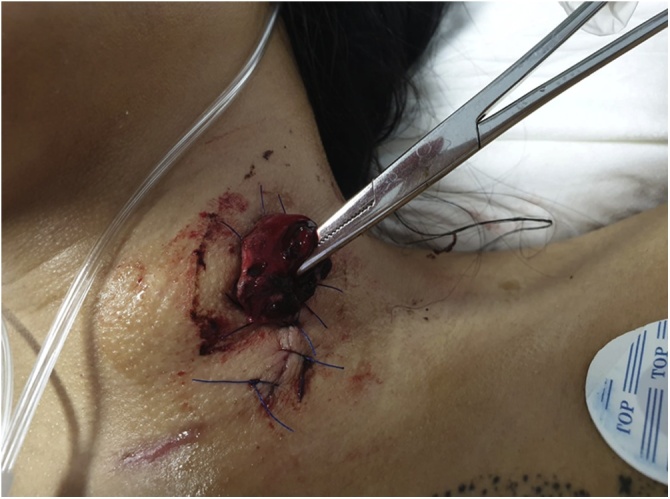
Cervical esophagostomy.

A 20 year – old transsexual male was admitted to the emergency department after voluntary ingestion of a moderate quantity of household cleaning product (WC NET GEL disincrostante- Ph 0.5, water soluble) attempting to commit suicide as a reply of a love quarrel. The patient rapidly developed respiratory distress, nausea, and vomiting, and comes to the emergency department 30 min after ingestion. For the dramatic respiratory distress and to protect the airway the patient was promptly intubated. Blood analysis showed: leukocytosis (24.50 × 10^3^/mm^3^), glucose 227 mg/dL, hematocrit 42.1 g/dL, bicarbonate 21 mmol/L, lipase 3853 U/L, amylase 312 U/L, LDH 473, procalcitonin 14.3 ng/mL. The coagulation parameters were normal. The initial treatment included intravenous fluids and antibiotics (piperacillin + tazobactam) administration. A chest/abdomen CT scan was performed in order to check eventual perforation and active source of bleeding: the result was a massive gastrectasia without signs of perforation. After, the UGI endoscopy revealed grade IIIB extensive necrotic lesions of the esophagus and the stomach with active bleeding from the gastric fundus not manageable endoscopically. For the hemodynamic instability and the progressive anemization, an emergent laparotomy was scheduled. During the operation for the evidence of gastro-duodenal-jejunal necrosis with 1 st stage gastric perforation we performed a total gastrectomy, pancreatoduodenectomy and resection of 40 cm of jejunum. The main pancreatic duct was sealed by the injection of glue and the parenchyma divided by ultrasonic device. The biliary flow was guarantee by hepaticojejunostomy. The esophagus was closed at the level of cardia, the secretion diverted using a Petzer sound, and a jejunostomy for enteral feeding was performed. The main operative time was 240 min and the blood loss 350 ml. The patient was transferred in ICU after surgery. He was estubated in PO day 1 and transferred on the ward in day 4. In PO day 10 the patient required a new surgical procedure for biliary peritonitis due to jejunum perforation at level of the cul-de-sac of the hepaticojejunostomy. The lesion was treated with raffia and concomitant placement of internal–external biliary drainage. The patient was managed extensively and explored endoscopically every month in order to evaluate the esophagus healing process. The nutritional support was constant, the weight remains stable all time and antibiotic therapy was continued for one month in absence of clinical/laboratory signs of infection. After three months the endoscopy showed, together with a critical stenosis, the presence of asymptomatic esophagobronchial fistula (Fig. 1, Fig. 2). We planned, after a meeting with thoracic surgery group, an open total esophagectomy (Fig. 3) and a muscular flap repair of bronchial fistula, 4 months after the ingestion. The post-operative course was uneventful and the patients continues the rehabilitation and the nutritional support through the enteral jejunal feeding. How to re establish the upper-gi continuity and to guarantee a physiologic food/fluid oral ingestion was largely discussed during internal department meeting. Six months after the initial operation, we underwent the digestive reconstruction with anterior retrosternal colonic esophagoplasty using distal transverse colon, transposed between cervical esophagus ad jejunum. Intestinal continuity was restored by colo-colic anastomosis. The operative time was 180 min without perioperative complications. The patient was discharged from the hospital three months after the last surgery (9 months after the ingestion) in good general condition with normal oral intake.

## 3 Discussion

Caustic ingestion represents a surgical emergency with a rate of morbidity and mortality of over 20%. A prompt diagnosis and supportive medical management are required to avoid severe immediate complications (perforation and necrosis) and achieve the goal of patient’s surviving. Initial management is directed towards a detailed and careful patient’s history (e.g. psychiatric background) and towards stabilization of life parameters; in this phase arterial blood gases could reveal life-threatening acid-base disorders and/or an increased lactic acid level. The first step is protect the airway. Vomiting and hematemesis can make difficult the initial management and there is a risk of severe acid-base imbalance. Early endoscopic examination in both adult and children is recommended and the Zargar endoscopic classification of caustic injuries is most commonly used. Ideally, UGI endoscopy should be performed within 12–24 hours from the ingestion of the caustic substance [8]. The major drawback of endoscopy is its inability to predict accurately transmural necrosis, which may expose patients to either futile surgery or inappropriate “watch and wait” management and risk of death. CT-scan, is important because suggest transmural digestive tract necrosis and evaluate eventual acute perforations. The management depends on the degree of the injury, as partial-thickness lesions (grades I, II) could be treated conservatively with fasting, intravenous fluids, and antibiotics. Full-thickness necrosis (grade III) may require immediate surgical intervention, especially in unstable patients or patients with intraperitoneal perforation [9]. Esophagectomy during the initial exploratory laparotomy is of little benefit, as it significantly increases the operating time, and control of contamination could be achieved with a cervical esophagostomy and intra-abdominal drainage of the mediastinum [10]. In addition, esophagectomy is an independent negative predictor of survival after emergency surgery. Decisively, every effort possible should be made to mature a feeding jejunostomy at the initial operation, to provide enteral feeding and avoid parenteral nutrition, which are known to prolong hospital stay, and increase the risk of infections. Multi-organ dysfunction and systemic inflammatory response syndrome (SIRS) must have resolved, and the patient must have good nutritional status, before one should plan a restorative procedure. In this case report restoration was performed six months after the index operation. Restoration of the GI tract is usually performed with a pedicle colonic graft, which is associated with satisfactory functional results and adequate durability. Transposition through the anterior mediastinum is the shortest route and provides the best functional outcome [11]. Another major issue to consider during the restorative procedure is whether to resect the esophagus; a strictured esophagus carries an 8% risk of developing malignancy over a 25- to 50-year period. Esophagectomy should definitely be performed in children and young adults with a long-life expectancy. However, in middle-aged adults or elderly patients, this issue should be a matter of sound assessment, and esophagectomy could be avoided. The large report was published by Cattan et al. reporting the experience in nine patients with a mean age of 45.8 years (range 36–62). The surgical approach required: total esophago-gastrectomy, pancreatoduodenectomy (4 cases), left splenopancreatectomy (2 cases), segmental colectomy (2 cases) and duodenostomy (1 case). Five patients required at least one reintervention in the postoperative period caused by colonic perforation, a necrotic-hemorrhagic pancreatitis and fistulized pancreatic abscess. Two patients died after the initial operation, one on day 17 of multisystem organ failure and the other on day 130 of pulmonary infection. The survivors underwent retrosternal ileocolonic esophagoplasty 4–8 months after initial operation. Three patients died after reconstructive surgery. Among the four survivors, three eat normally and one patient has dysphagia. In summary this experience report how hard and life-threatening is the caustic ingestion: mortality rate for initial operation 22%; mortality rate after reconstruction 43%; survival rate 44% [12]. The caustic ingestion remains at present a very complex complication, hard to treat and requiring large efforts and multi-specialty experience.

## 4 Conclusions

Our case report demonstrated how hard is manage a patient after caustic ingestion: it required a large experience by the emergency surgery, by the anesthesiologist, by the endoscopist and by all the hospital that should be ready to manage every possible complication for months (9 months in our case). Refer the patient to large and experienced hospital represents the main goal to guarantee a surviving chance.

## Declaration of Competing Interest

Nothing to declare.

## Funding

Any source of funding was involved in this study.

## Ethical approval

The ethical approval was obtained by the Internal Surgical Department.

## Consent

Written informed consent was not obtained from the patient. The head of our medical team has taken responsibility that exhaustive attempts have been made to contact the family and that the paper has been sufficiently anonymised not to cause harm to the patient or their family. A copy of a signed document stating this is available for review by the Editor-in-Chief of this journal on request.

## Author contribution

Maggioni Cristina: Study concept, writing the paper, operate the patient.

Voltolini Luca: Data analysis.

Bongiolatti Stefano: Study concept, contribution to patient’s operation.

Cianchi Fabio: Supervising, study concept.

Coratti Francesco: Study concept and design, data interpretation, operate the patient.

## Registration of research studies

NA.

## Guarantor

Cristina Maggioni, MD represents the paper guarantor.

## Provenance and peer review

Not commissioned, externally peer-reviewed.
